# Source Indication and Geochemical Significance of Sedimentary Organic Matters from the Xisha Area, the South China Sea

**DOI:** 10.3390/molecules26226808

**Published:** 2021-11-11

**Authors:** Yan Li, Qian-Zhi Zhou, Xiang-Po Xu, Yun-Xin Fang, Jin-Zhong Liu, Jiang-Hai Wang

**Affiliations:** 1 Guangdong Provincial Key Laboratory of Marine Resources and Coastal Engineering, School of Marine Sciences, Sun Yat-sen University, Zhuhai 519082, China; liyan255@mail.sysu.edu.cn (Y.L.); zhouqzhi@mail2.sysu.edu.cn (Q.-Z.Z.); xuxp35@mail2.sysu.edu.cn (X.-P.X.); 2 Guangzhou Marine Geological Survey, Guangzhou 510760, China; 3 State Key Laboratory of Organic Geochemistry, Guangzhou Institute of Geochemistry, Chinese Academy of Sciences, Guangzhou 510640, China; liujinz@gig.ac.cn

**Keywords:** source indication, sedimentary organic matter, biomarkers, petroleum hydrocarbons, Xisha area, South China Sea

## Abstract

Although various geochemical and geophysical investigations have already indicated a great resource potential in the Xisha area of the South China Sea, the origin of organic matter and molecular evidence for tracing the migration of hydrocarbons from deep petroleum reservoirs are still lacking. In this study, systematic organic geochemical analyses, including bulk organic matter parameters and lipid biomarkers were performed for deep sediments from two cores. The C/N ratios and δ^13^C and δ^15^N values of organic matter in most of the samples, together with the maxima of short-chain *n*-alkanoic acids and mid-chain *n*-alkanols, high abundances of monounsaturated fatty acids C_18:1ω9_ and C_22:1ω13_, jointly indicate the dominance of marine organic matter. *n*-Alkanes in sediments from core GMGS4-XH-W06B are characterized by small unresolved complex mixture (UCMs) humps, high odd/even predominance (OEP) and carbon preference index (CPI) values, clearly exhibiting characteristics of modern sediments. However, the sediments for core GMGS4-XH-W03B are featured with big UCMs, associated with OEP and CPI values around 1.0, showing signatures of petroleum hydrocarbons from high maturity sources. Considering the geologic background, the biomarker signatures are solid evidence for indicating the existence of underlying petroleum reservoirs, and may provide the valuable information for assessing the hydrocarbon resources in the Xisha area.

## 1. Introduction

The South China Sea (SCS) is located at the confluence of the Pacific, Eurasian, and Indian-Australian plates, and was formed during the late Jurassic-early Cretaceous [1]. It harbors numerous natural gas hydrates and conventional gas/oil reservoirs [2,3,4]. In light of the great gas hydrate and petroleum/gas reservoir potential, as well as the significant environmental effects (e.g., global warming and petroleum/gas exploration), the SCS has attracted the increasing attention of marine geologists and geochemists [5,6,7].

Recently, many oil/gas fields have been found in the SCS, such as the Beibu Gulf Basin, Pearl River Mouth Basin and Wan’An Basin [8]. The prospecting areas of gas hydrates, such as Dongsha, Xisha, Qiongdongnan and Shenhu, were firstly determined via the extension of bottom-simulating reflectors (BSRs) [9,10]. After several hydrate survey expeditions, some hydrate-bearing areas, e.g., Dongsha and Shenhu, have further been confirmed [7,10,11]. In addition, numerous cold seeps have also been discovered in the Dongsha and Shenhu areas, indicating the hydrocarbon seepage and occurrence of underlying gas hydrates [5]. In these areas, many studies have been conducted, mainly including geochemistry, geophysics, numerical simulation, sediment fabrics, microbial diversity, and biomarkers [7,11,12,13,14,15,16,17,18,19]. Notably, the contribution of petroleum hydrocarbons from deep oil reservoirs in these areas was revealed by the analyses of 16S rRNA and biomarkers [7,11,19].

The Xisha area, adjacent to many oil and gas fields, is located in the northern slope of the SCS. This area is characterized by high sedimentation rates and steep geothermal gradients. As a result, overpressures occur in sedimentary strata and the maturity of their organic matters in this area was enhanced, thereby a large amount of hydrocarbons were formed and migrated toward the seabed [20]. To our knowledge, no gas hydrates have been found in the Xisha area, despite the BSRs, porewater characteristics and the occurrence of pockmarks suggest a great resource potential [20,21,22]. During the last decades, numerous studies have been conducted in this area, but their attention was mainly focused on the formation of carbonates and their paleoclimate evolution [23,24], and no direct investigation on the organic geochemical evidence related to a source indication for verifying the existence of petroleum/gas reservoirs and gas hydrates in the Xisha area has been reported. Therefore, we still do not clearly know the characteristics of sedimentary organic matter in this area, which may provide important information for identifying gas hydrate and petroleum/gas reservoirs.

**Figure 1:**
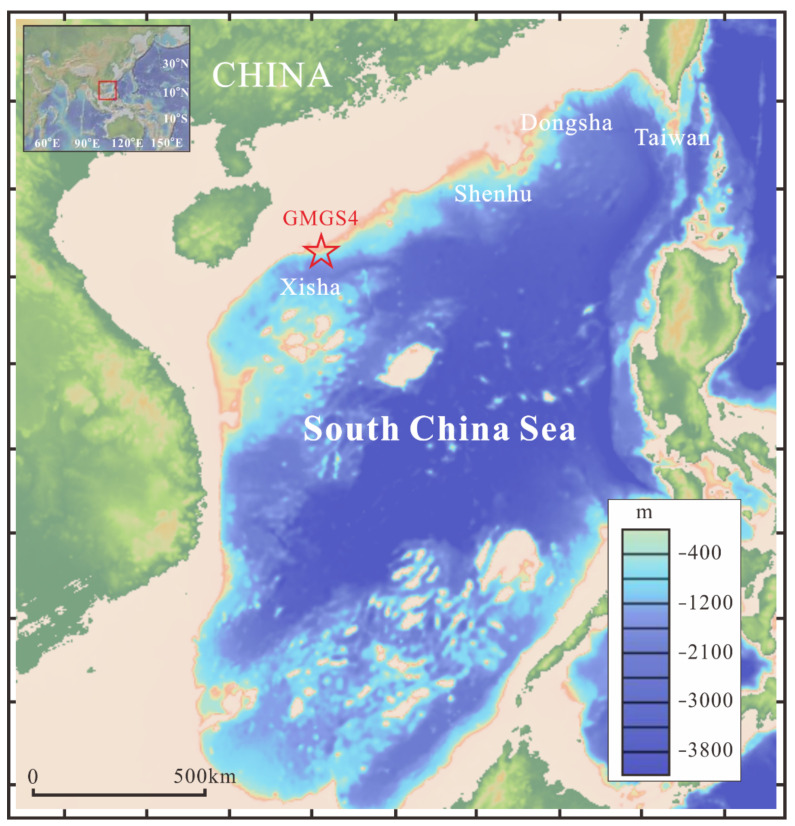
Map illustrating the locations of sediment cores GMGS4-XH-W03B and GMGS4-XH-W06B from the Xisha area, the South China Sea (SCS) (Adapted with permission from ref. [5]. Copyright 2018 Clearance Center).

**Figure 2:**
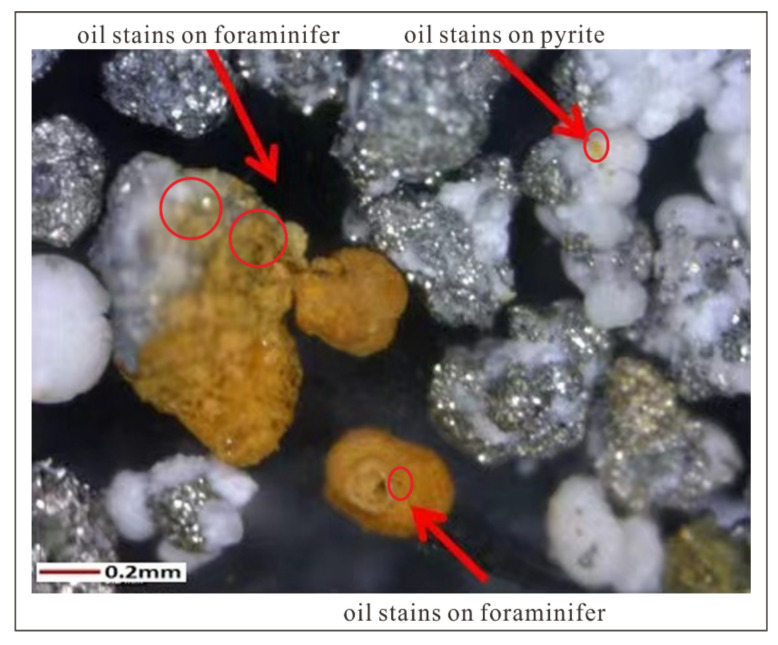
Oil stains in sample 2 from core GMGS4-XH-W03B. Red arrows reflect the direction indicated by the comment text; red circles suggest the locations of oil stains.

The information of sedimentary organic matter, such as bulk organic matter parameters and lipid biomarker signatures, may provide insight into the original source inputs [25,26,27,28]. It is notable that pollution is correlated with organic matters as reported from previous studies [29,30,31]. In the present work, the deep sediments from two cores (GMGS4-XH-W03B and GMGS4-XH-W06B) from gas hydrate drilling expeditions in the Xisha area of the SCS were selected for systematically performing organic geochemical analysis (Table 1; Figure 1), including total organic carbon (TOC) and total nitrogen (TN) contents, C/N ratios, and δ^13^C and δ^15^N values, as well as biomarker (i.e., *n*-alkanes, *n*-alkanols, steroids, and fatty acids) distribution patterns and sedimentary organic matter compositions. Notably, oil stains were observed in sample 2 from core GMGS4-XH-W03B (Figure 2). The purpose of this study is to compare the origin of the organic matter and determine oil/gas seepage signatures for evaluating the potentials of underlying gas hydrates and conventional oil/gas reservoirs.

## 2. Results

**Figure 3:**
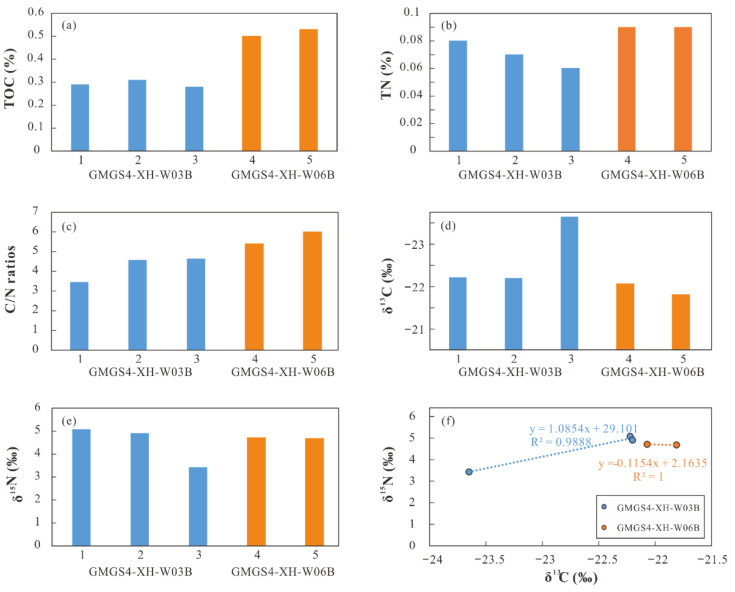
(**a**) TOC contents; (**b**) TN contents; (**c**) C/N ratios; (**d**) δ^13^C values; (**e**) δ^15^N values; and (**f**) cross-plot of the δ^13^C and δ^15^N values in sediments.

The TOC and TN contents, C/N ratios, and δ^13^C and δ^15^N values are presented in Table 2 and Figure 3. The TOC contents of the samples from cores GMGS4-XH-W03B and GMGS4-XH-W06B are about 0.30% and 0.50%, respectively. The TN contents in the samples from two cores range from 0.06% to 0.09%. The atomic C/N ratios in the samples from core GMGS4-XH-W03B are at the interval of 3.46 to 4.65; while those for core GMGS4-XH-W06B range from 5.40 to 6.00. The difference between the C/N ratios of both core samples is mainly due to their TOC contents rather than TN contents. The δ^13^C and δ^15^N values of the samples from core GMGS4-XH-W03B are in the range of −23.65‰ to −22.20‰ and 3.43‰ to 5.08‰, respectively; whereas those for core GMGS4-XH-W06B are from −22.07‰ to −21.81‰ and 4.68‰ to 4.71‰, respectively. As a whole, the TOC and TN contents and C/N ratios in the studied samples are nearly similar, but those in the samples from core GMGS4-XH-W06B are slightly higher. Except sample 3 in core GMGS4-XH-W03B, both δ^13^C and δ^15^N values are comparable in the two cores. In addition, a strong positive correlation between the δ^13^C and δ^15^N values exhibits for our data set (Figure 3f). For the bulk organic carbon/nitrogen and their stable isotope values, it is interesting that there is no difference in the sample with oil stains.

**Figure 4:**
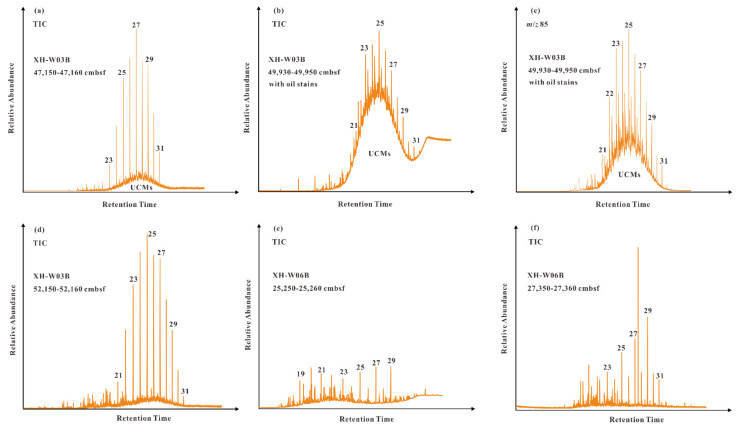
GC-MS chromatograms showing the distribution of *n*-alkanes in sediments from (**a**–**d**) core GMGS4-XH-W03B and (**e**,**f**) core GMGS4-XH-W06B. (**a**,**b**) and (**d**–**f**) represent total ions chromatograms; (**c**) represent chromatogram of fragment ion *m*/*z* 85. Arabic numerals indicate the numbers of carbon atoms.

The signatures of *n*-alkanes in the samples from two cores display similarities and differences to some extent (Table 2; Figure 4). As a whole, the *n*-alkanes in all samples are mainly distributed in the range of *n*-C_17_ to *n*-C_31_; short-chain *n*-alkanes are relatively low in abundance (the C_21−_/C_22+_ values less than 0.28). Notably, the samples from core GMGS4-XH-W03B are primarily characterized by the dominance of medium-chain *n*-alkanes with a maximum at *n*-C_25_; and the humps of unresolved complex mixture (UCMs) occur in most of them, particularly the occurrence of big UCMs humps in the sample with oil stains. While *n*-alkanes in the samples from core GMGS4-XH-W06B are generally in low abundances with small UCMs humps, and dominated by long-chain *n*-alkanes with a maximum at *n*-C_27_ and *n*-C_29_. The carbon preference indices (CPI) and odd/even predominance ratios (OEP, *i* = 23) are around 1.0 in the samples from core GMGS4-XH-W03B; while those for the samples from core GMGS4-XH-W06B are at the interval of 2.33 to 2.92 and 1.97 to 1.98, respectively, with the odd-over-even carbon number predominance. The biomarkers, such as pristane, phytane, steranes and hopanes, are useful to provide the significant information on the source inputs and thermal maturity, and may hold clues on the oil/source correlation since they can be steadily detected from oil/gas reservoirs [27]. Unfortunately, pristane and phytane are usually below detection lines, and only recognized in a few samples but with low abundances. It is noteworthy that terpanes and steranes are undetectable, which is similar to the previous result that the conventional GC-MS could not clearly identify these biomarkers due to their low abundances [7]. Therefore, indicators of thermal maturity such as Ts/(Ts + Tm) and C_29_ 20S/(20S + 20R) sterane ratios cannot be acquired in this study.

**Figure 5:**
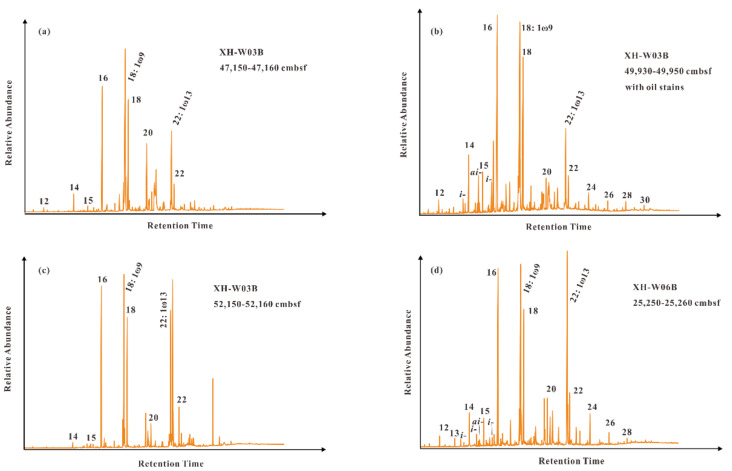
GC-MS chromatograms showing the distribution of fatty acids in sediments from (**a**–**c**) core GMGS4-XH-W03B and (**d**) core GMGS4-XH-W06B. Arabic numerals denote the numbers of carbon atoms. 18:1ω9 and 22:1ω13 refer to C_18:1ω9_ and C_22:1ω13_ mono-unsaturated fatty acids. The symbols *i-* and *ai-* represent *iso*-C_14:0_, *iso*-C_15:0_, *anteiso*-C_15:0_, and *iso*-C_16:0_, respectively.

The *n*-alkanoic acids mainly range from C_12_ to C_22_ in most of the samples and long-chain *n*-alkanoic acids (C_24_ to C_30_) only occur in a few samples (Table 2; Figure 5). Overall, the distribution of *n*-alkanoic acids in the studied samples is similar, and characterized by the dominance of short-chain *n*-alkanoic acids with a maximum at C_16_ and significant even-over-odd carbon number predominance. Unsaturated fatty acids such as C_18:1ω9_ and C_22:1ω13_ generally show extremely high abundances, even higher than those of their adjacent saturated fatty acids. Branched fatty acids, including *iso*-C_14:0_, *iso*-C_15:0_, *anteiso*-C_15:0_, and *iso*-C_16:0_, are detected in sample 2 and sample 4. Notably, *iso*-C_15:0_ and *anteiso*-C_15:0_ are considered as two specific biomarkers for sulfate reducing bacteria [32]; and the calculated *i*-C_15:0_ + *ai*-C_15:0_/C_15:0_ ratio in sample 2 with oil stains and sample 4 are 1.1 and 0.4, respectively (Table 2; Figure 5).

**Figure 6:**
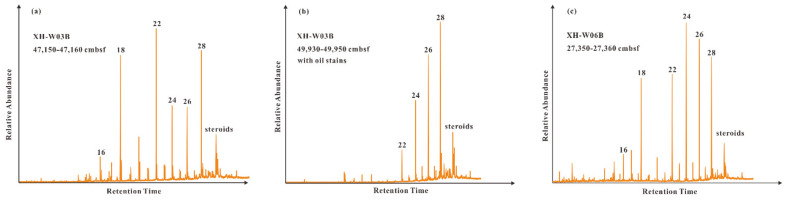
GC-MS chromatograms showing the distribution of *n*-alkanols and steroids in sediments from (**a**,**b**) core GMGS4-XH-W03B and (**c**) core GMGS4-XH-W06B. Arabic numerals indicate the numbers of carbon atoms.

The *n*-alkanols in all samples generally range from C_16_ to C_28_, with a maximum at C_22_, C_24_, and C_28_ (Table 2; Figure 6). Similar to *n*-alkanoic acids, the *n*-alkanols also show a significant even-over-odd carbon number predominance (Figure 6). Meanwhile, a series of steroids (sterols and stanols) were detected in all the samples but with relatively low abundances.

## 3. Discussion

### 3.1. Source Indication of Organic Matter in Sediments

The source of organic matter in marine sediments has been frequently investigated using bulk organic matter parameters [28,33,34,35]. Multiple indices have been applied to track the source of organic matter, specifically the C/N ratios together with δ^13^C and δ^15^N values are widely used to distinguish between terrestrial and marine sources of sedimentary organic matter [34,36]. For instance, the C/N ratios within 4–9 indicate marine algae as the main source of organic matter; while those higher than 20 are taken as an indicator for evaluating the contribution of terrestrial organic matter [33,37]. Therefore, the C/N ratios (3.46–6.00) indicate that the main source in the studied sediments is marine organic matter. Notably, the C/N ratios are comparable to those reported previously for the hydrate-bearing sediments (5.79–6.75) in the Taixinan Basin of the SCS [19], while are obviously lower than those in the surface sediments (7.3–11.0) from the northern SCS [38].

**Figure 7:**
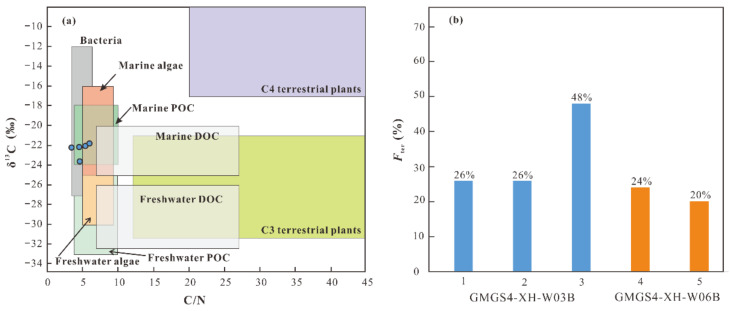
(**a**) Source indication inferred from the δ^13^C values and C/N ratios of organic matter. Adapted with permission from ref. [28]. Copyright 2006 Clearance Center; (**b**) calculated percentages of terrestrial organic matter (*F*_ter_) in sediments. POC and DOC denote particulate organic carbon and dissolved organic carbon, respectively.

In contrast to the C/N ratios, the δ^13^C values may indicate the source of sedimentary organic matter more accurately [39]. The δ^13^C values are commonly applied to identify between terrestrial and marine sources of organic matter in marine environments. Generally, the δ^13^C values of marine organic matter are at the interval of −22‰ to −19‰; whereas terrestrial organic matter are isotopically lighter than its marine counterpart, typically within the range of −28‰ to −26‰ [40,41]. Therefore, the δ^13^C values (−23.65‰ to −21.81‰) suggest that the sedimentary organic matter in this study are primarily of marine origin but with a minor contribution of terrestrial plants. Furthermore, it has been reported that the plot between C/N ratios and δ^13^C values is an effective tool for qualitatively analyzing the sources of organic matter [28]. As illustrated in Figure 7a, the C/NFigure ratios and δ^13^C values in the studied sediments fall in the mixed range of different organic matter derived from marine algae, marine particulate organic carbon (POC), and bacteria.

To distinguish the sources of organic matters more accurately, a two end-member model was employed in this study for quantitatively assessing the contribution of terrestrial and marine organic matter according to their δ^13^C values [34,42]:

*F*_ter_ (%) = [δ^13^C_sam_ − δ^13^C_mar)_]/[δ^13^C_ter_ − δ^13^C_mar_] × 100 (1)

where *F*_ter_ (%) and δ^13^C_sam_ represent the percentages of terrestrial organic matter and measured δ^13^C values of samples, respectively; and δ^13^C_mar_ and δ^13^C_ter_ represent the marine and terrestrial end members, respectively.

For the studied area, we adopted −27‰ and −20.5‰ as the terrestrial and marine end-member values respectively, as reported by Hu et al. [34]. The calculated relative percentages of terrestrial organic matter in the studied sediments are presented in Table 2 and Figure 7b. The estimated result (the ratios at the interval of 20% to 48%) indicates that the contribution of terrestrial organic matter is relatively low (except sample 3 from core GMGS4-XH-W03B), which is consistent with the conclusion mentioned above.

Likewise, the δ^15^N values vary significantly among different sources, and are also used as a proxy of the provenance of organic matter in marine sediments [42]. It is well-known that terrestrial organic matter show a lower δ^15^N value than that of marine organic matter, because marine planktons generally use isotopically heavier nitrates as their nitrogen source in comparison with terrestrial plants. For instance, the typical δ^15^N values of terrestrial vascular plants range from −5‰ to 18‰ with the mean value of ~3‰ [34]. Liu [35] further reported that the δ^15^N values of terrestrial C3 and C4 plants were in the ranges of 5–18‰ and 3–6‰, respectively. As we know, the average δ^15^N value of marine nitrate is about 5‰ [43]; the average δ^15^N value of marine POC is in the range of 3–12‰ with an average value of 6‰ [34]; and the δ^15^N values of organic nitrogen produced by nitrogen-fixing microorganisms and phytoplankton are at the intervals of 0–2‰ and 5.5–8.5‰, respectively [44,45]. In addition, the δ^15^N values of sediments from the subtropical Pearl River estuary and adjacent shelf, the South China range from 3.9‰ to 6.4‰ [34], which is comparable to the obtained values (3.43–5.08‰) in this study. Overall, in combination with the C/N ratios and δ^13^C values, the δ^15^N values also reflect the dominance of marine organic matter. Meanwhile, the strong positive correlation between the δ^13^C and δ^15^N values in the studied samples reflects that there might be a high proportion of labile organic matter in freshly supplied marine organic material [46].

### 3.2. Origin of Lipid Biomarkers in Sediments

As mentioned above, the C/N ratios and δ^13^C and δ^15^N values have long been recognized for its important role in tracing the source input of sedimentary organic matter. However, the source of organic matter in marine sediments is not well understood due to the complexity of source inputs and biogeochemical processes such as biodegradation [47]. Therefore, accurately tracing the source input of sedimentary organic matter has long been a research focus. In fact, biomarkers originated from multiple source inputs are of indispensable advantage in precisely identifying the origin and provide complementary data for identifying the source input, because they are chemically stable from the bio-precursor molecules, even after decomposition and diagenesis [27]. In addition, the application of multi-biomarker molecules (i.e., *n*-alkanes, *n*-alkanols, steroids and fatty acids) with mutually confirmable information is capable of stricter constraining the sources, in particular for marine environments with complex source inputs [19].

On the whole, the distribution patterns of *n*-alkanoic acids and *n*-alkanols in two core samples are basically similar, but *n*-alkanes are obviously different between cores GMGS4-XH-W03B and GMGS4-XH-W06B. In most of the samples, the dominance of medium-chain *n*-alkanes with a maximum at *n-*C_25_ reflects a large source input of aquatic plants [25]. The predominance of long-chain *n*-alkanes with maxima at *n-*C_27_ and *n-*C_29_ suggests that terrestrial higher plants may be dominant [48]. It should be noted that algae (i.e., *Botryococcus braunii* race A) can also produce odd numbered long-chain *n*-alkanes [26,49]. In this study, UCMs were detected in most of the sediments from core GMGS4-XH-W03B, especially sample 2 with oil stains, implying that these sediments were likely contaminated with petroleum hydrocarbons [6,27,50]. It is notable that UCMs are ubiquitous in microbially degraded crude oil, and commonly ascribed to the occurrence of hydrocarbons related with oil pollution or petroleum seepage [6,27]. In addition, UCMs have frequently been observed in authigenic seep carbonates from the Gulf of Mexico and the SCS where oil seepage occurred [17,51]. Furthermore, *n*-alkane parameters, including CPI and OEP ratios are generally about 1.0 in the samples from core GMGS4-XH-W03B, implying a relatively high thermal maturity, because the petroleum source of organic matter input or hydrocarbon leakage from underlying high-maturity source formations generally exhibits CPI and OEP ratios around 1.0 [52]; whereas the CPI (2.33 to 2.92) and OEP (1.97 to 1.98) ratios, together with the odd-over-even predominance and small UCMs in the sediments from core GMGS4-XH-W06B are characteristics of modern sediments. Notably, our team also conducted organic geochemical analysis in gas hydrate sediments from the Shenhu area [7] and the Taixinan area [19,53] of the SCS. In our previous studies, we reported the wide occurrence of UCMs and OEP values around 1.0 in both shallow and deeply-buried hydrate-bearing sediments from the SCS [7,19,53]. Adopting the technique of GC×GC-TOFMS, 25-norhopane, pristane, phytane and hopanes were further identified [7]. Unfortunately, terpanes and steranes are undetectable in this study, but the presence of UCMs, associated with CPI and OEP ratios, provides important evidence for supporting the source input of petroleum hydrocarbons in the sediments from core GMGS4-XH-W03B.

For *n*-alkanoic acids, the samples were generally characterized by the dominance of short-chain *n*-alkanoic acids with a maximum at C_16_ and the significant even-over-odd carbon number predominance, showing the low concentrations of long-chain *n*-alkanoic acids. Short-chain *n*-alkanoic acids are nonspecific biomarkers for source identification since their widespread occurrence in marine fauna, bacteria, microalgae, and higher plants [54,55], however the absence or extremely low concentrations of long-chain *n*-alkanoic acids indicates an insignificant contribution of terrestrial plants. Monounsaturated fatty acids, dominated by C_18:1ω9_ (ω represents the number of double bonds) and C_22:1ω13_), were detected with a relatively high abundance second only to the maximum component (C_16_). These characteristics indicate a large input of organic matters from microalgae and zooplanktons [56,57,58,59]. Further, branched fatty acids, including *iso*-C_14:0_, *iso*-C_15:0_, *anteiso*-C_15:0_, and *iso*-C_16:0_ are discriminated from in two of these samples, especially sample 2 with oil stains. Generally, these compounds are considered to be mainly bacterial sources [32,60]; and the relatively high abundances of *iso*-C_15:0_ and *anteiso*-C_15:0 in the sample with oil stains reflect that_ sulfate reducing bacteria may primarily thrive on petroleum hydrocarbons [32,61,62,63].

In this study, the *n*-alkanols in all samples generally range from C_16_ to C_28_, with the maximum at C_22_, C_24_, and C_28_. Usually, the high abundances of even-odd-distributed long-chain *n*-alcohols (i.e., C_24_ and C_28_) seem to indicate a contribution of organic matter from terrestrial higher plants [50,64], whereas the short-chain *n*-alcohols (<C_22_), in comparable abundances to the long-chain *n*-alcohols, are considered to be derived from marine planktons, diatoms, and bacteria [65,66]. Steroids (sterols and stanols), as particularly useful biomarkers for assessing the source of organic matter in aquatic ecosystems [67,68], are detected in all the samples but with relatively low abundances. Unfortunately, no specific sterols and stanols were identified due to their low abundances and co-elution with adjacent compounds. Overall, the composition and distribution of various biomarkers suggest that the original source inputs of organic matters in the studied samples are multiple, including marine planktons, terrestrial plants, and bacteria. Notably, based on the occurrence of oil stains in sample 2 and big UCMs as well as the CPI and OEP values in all samples from core GMGS4-XH-W03B, the petroleum hydrocarbons migrated from underlying high-maturity reservoirs may be a source input of organic matter. In summary, the bulk organic geochemical parameters and various lipid biomarkers jointly indicate that marine organic matter are a dominant source input for the autochthonous organic matter; and the discrepancy in the source interpretation among *n*-alkanes, fatty acids (i.e., *n*-alkanoic acids and branched fatty acids), and *n*-alkanols may be due to multiple possible sources for some molecules.

### 3.3. Geochemical Implications on Underlying Petroleum/Gas Reservoirs

Upward migration of petroleum hydrocarbons is generally regarded as a window for tracing deep petroleum/gas reservoirs. As discussed in Section 3.2, the evident biomarker signatures (including the CPI and OEP values around 1.0 and occurrence of big UCMs) associated with petroleum hydrocarbons were identified from core GMGS4-XH-W03B. As reported previously that the Xisha area is adjacent to many oil/gas fields, including the Pearl River Mouth Basin, Beibuwan Basin, Yinggehai Basin, Qiongdongnan Basin, and Taixinan Basin [2,3,8]. In terms of the geological and tectonic evolution in these areas, they have a close relationship and share the similar characteristics [10]. In addition, it is widely acknowledged that the high-quality source rocks for generating hydrocarbons formed in the Eocene–Oligocene rift in the Xisha area, in particular the sedimentary sequences characterized by lacustrine and neritic mudstone facies and coastal plain coal-bearing layers [21]. Further, the high sedimentation rate and geothermal gradient (72–89 °C/km) are extremely favorable for the maturation of organic matters and hydrocarbon generation. The discoveries of BSRs [9] and mega pockmarks (1000–2500 m in diameter and 60–140 m in depth) in this region [20,21], reflect the occurrence of structures associated with the underlying fluid migration [69]. Apparently, the occurrence of these structures such as unconformities, active faults, and diapirs supports the inference that petroleum hydrocarbons were migrated from underlying oil/gas reservoirs.

As a whole, the organic geochemical results, mainly including biomarker distribution patterns and compositions in sediments from core GMGS4-XH-W03B provide a new clue for evaluating the potential of underlying petroleum reservoirs in the Xisha area. The geological backgrounds and tectonic activities also confirmed the possibility of the generation and migration of petroleum hydrocarbons in this area. Similarly, the molecular evidence reflecting the contribution of petroleum hydrocarbons from deep oil reservoirs was reported in the Shenhu area [7] and Taixinan Basin [11,19,53]. These results reveal that the migration of petroleum hydrocarbons is a common phenomenon in the SCS; and the studies on the bulk organic matter and lipid biomarkers are reliable approaches for seeking the migration of petroleum hydrocarbons from deep petroleum/gas reservoirs. Unfortunately, our newly-obtained biomarker evidence is still unlikely to confirm whether gas hydrates exist or not, despite gas hydrates and conventional petroleum/gas reservoirs generally coexist in the SCS. It is notable that the sulfate-methane interface (SMI) depth can reflect the existence of underlying gas hydrates, i.e., a relatively shallow SMI depth (generally < 10 mbsf) above hydrate deposits [70]. Considering the SMI depths of 70 m and 150 m in these cores, together with the absence of the specific biomarkers associated with methanotrophic archaea, it is not easy for gas hydrate systems to develop in the Xisha area.

## 4. Materials and Methods

### 4.1. Sampling

Two sediment cores for gas hydrate drilling expeditions (GMGS4) were collected in 2016 from the Xisha area (Figure 1) at the stations GMGS4-XH-W03B (1777 m water depth; 72 °C/km geothermal gradients) and GMGS4-XH-W06B (1923 m water depth; 89 °C/km geothermal gradients). The depths of sulfate-methane interface (SMI) for sediment cores GMGS4-XH-W03B and GMGS4-XH-W06B are 70 m and 150 m, respectively. Sediments from the two cores are yellowish gray, unconsolidated, and fine-grained silty clay. The samples were transported to the laboratory and stored at −20 °C until analysis. In this study, we selected 5 representative samples from deep sediments for organic geochemical analysis, including one sample with oil stains (i.e., sample 2) (Table 1).

### 4.2. Experimental Analysis

The TOC and TN contents, C/N ratios, and δ^13^C and δ^15^N values of sedimentary organic matters were measured by a CE EA1112 C/N elemental analyzer (CE Instruments, Wigan, UK)-Delta Plus XL stable isotope ratio mass spectrometry (Finnigan, Thermo Scientific, Waltham, MA, USA). Before analysis, carbonates were removed with 3 M HCl, then washed with distilled water, and finally freeze-dried. Prior to the analysis, a standard (acetanilide) was measured for quality control, with the instrument precision deviations of both TOC and TN contents less than/equal to 0.10% abs. The δ^13^C and δ^15^N values were reported in the delta (δ) notation relative to the V-PDB standard and atmospheric N_2_, respectively, with the corresponding standard deviations within ±0.05‰ and ±0.14‰ respectively over the analytical course.

Freeze-dried samples were ground to less than 120 mesh; and about 20 g were Soxhlet- extracted with a mixture of dichloromethane/methanol (3:1, *v*/*v*) (48 °C, 72 h). After removing the extracted elemental sulfur, the fractions were dried with sodium sulfate and concentrated; and then the total lipid extracts were saponified with KOH/methanol solution (1 M, 70 °C, 2 h). The neutral fractions were extracted with *n*-hexane for 3–5 times; and fatty acids were extracted after acidification (HCl, pH 1–2). The neutral fractions were then subjected to column chromatography for obtaining saturated hydrocarbons, aromatics, and fatty alcohols using *n*-hexane, *n*-alkanes/dichloromethane (4:1; *v*/*v*) and dichloromethane/methanol (1:1; *v*/*v*) as elutions, respectively. The fatty alcohols and fatty acids were further converted into trimethylsiloxyl derivatives and fatty acids methyl esters (FAMEs) following a previous reference [71].

Gas chromatography-mass spectrometry (GC-MS) analysis for saturated hydrocarbons, fatty alcohols, and fatty acids were performed on an Agilent 7890A gas chromatograph coupled with a 5975C mass spectrometer (Agilent Technologies, Palo Alto, CA, USA). Separation was achieved with the HP-5 MS fused a silica capillary column (30 m × 0.25 mm i.d. × 0.25 μm). Helium was used as the carrier gas with a flow rate of 1.0 mL/min. The injector and detector temperatures were set at 290 °C and 300 °C, respectively; and 2 μL of each concentrated sample were injected in the splitless mode. The oven temperature was initially set at 80 °C (held for 5 min); and then programmed at 3 °C/min to 290 °C (held for 20 min). The ion source was operated in the electron impact (EI) mode at 70 eV; and the full scan mass spectra were adopted. The compound assignment was compared to the NIST11 library of mass spectra as well as with the published data.

## 5. Conclusions

The features of C/N ratios, δ^13^C and δ^15^N values, and biomarker distributions (*n*-alkanes, *n*-alkanols, steroids and fatty acids) in deep sediments from cores GMGS4-XH-W03B and GMGS4-XH-W06B in the Xisha area, the SCS are investigated. The bulk organic C/N ratios and stable C-N isotope values (δ^13^C and δ^15^N) for the studied samples indicate a dominance of marine algae and bacteria inputs but a low input of terrestrial plants. The source indication from biomarker signatures is generally in accord with the bulk geochemical parameters, showing an immature source with marine organic matter dominated, i.e., the small UCMs, high OEP and CPI values of *n*-alkanes, dominance of short-chain *n*-alkanoic acids (C_16_–C_22_) and mid-/long-chain *n*-alkanols (C_22_–C_28_) with a significant even-over-odd carbon number predominance, high abundance of monounsaturated fatty acids C_18:1ω9_ and C_22:1ω13_, and occurrence of steroids. However, the *n*-alkanes in the sediments from core GMGS4-XH-W03B exhibit mature petroleum characteristics, i.e., big UCMs (especially sample 2 with oil stains) and OEP and CPI values around 1.0. Considering the high sedimentary rate and geothermal gradient as well as tectonic activities, the migration of petroleum hydrocarbons from underlying oil/gas reservoirs are plausible. Overall, our results may provide valuable information for evaluating the potential of hydrocarbon resources and reflecting the existence of underlying petroleum reservoirs in the Xisha area, the SCS.

## Figures and Tables

**Table 1:** Sample information.

Stations	Water Depth (m)	Geothermal Gradients (°C/km)	SMI Depth (m)	Sample ID	Depth (cmbsf)
GMGS4-XH-W03B	1777	72	70	1	47,150–47,160
				2	49,933–49,946
				3	52,150–52,160
GMGS4-XH-W06B	1923	89	150	4	25,250–25,260
				5	27,350–27,360

Notes: SMI represents the sulfate-methane interface.

**Table 2:** Analytical results of the bulk geochemical parameters and biomarker ratios for sediment cores.

Stations	GMGS4-XH-W03B			GMGS4-XH-W06B	
**Sample ID**	1	2	3	4	5
TOC (%)	0.29	0.31	0.28	0.50	0.53
TN (%)	0.08	0.07	0.06	0.09	0.09
C/N ratios	3.46	4.58	4.65	5.40	6.00
δ^13^C (‰)	−22.22	−22.20	−23.65	−22.07	−21.81
δ^15^N (‰)	5.08	4.91	3.43	4.71	4.68
*F*_ter_ (%)	26	26	48	24	20
***n*-Alkanes**					
Carbon ranges	C_21_–C_31_	C_17_–C_32_	C_21_–C_31_	C_17_–C_29_	C_23_–C_31_
C*x*	C_27_	C_25_	C_25_	C_27_	C_29_
Size of UCMs hump	big	big	small	small	small
CPI_24–33_	1.15	1.35	1.14	2.92	2.33
OEP(*i* = 23)	1.08	1.18	1.05	1.98	1.97
C_21−_/C_22+_	0.01	0.08	0.02	0.28	0.00
**Fatty acids**					
Carbon ranges	C_12_–C_22_	C_12_–C_30_	C_14_–C_22_	C_12_–C_28_	n.a
C*x*	C_16_	C_16_	C_16_	C_16_	n.a
*i*-C_15:0_ + *ai*-C_15:0_/C_15:0_	n.d	1.1	n.d	0.4	n.a
***n*-Alkanols**					
Carbon ranges	C_16_–C_28_	C_18_–C_28_	n.a	n.a	C_16_–C_28_
C*x*	C_22_	C_28_	n.a	n.a	C_24_

Notes: *F*_ter_ (%) represents the percent contribution of terrestrial organic matters. C*_x_* = Dominant carbon number; CPI_25–33_ = 1/2 [Σ(C_25_ − C_33_)_odd_/Σ(C_24_ − C_32_)_even_ + Σ(C_25_ − C_33_)_odd_/Σ(C_26_ − C_34_)_even_]; OEP = [(C*_i_* + 6C*_i_*_+2_ + C*_i_*_+4_)/(4C*_i_*_+1_ + 4C*_i_*_+3_)]^(^^−1)*i*+1^, *i* = 23; n.d., not detected; and n.a., not acquired.

## Data Availability

The data used to support the findings of this study are available from the corresponding author upon request.

## Sample Availability

Samples of the compounds are not available from the authors.
